# Targeted Radionuclide Therapy

**DOI:** 10.3390/cancers3043838

**Published:** 2011-10-11

**Authors:** Devrim Ersahin, Indukala Doddamane, David Cheng

**Affiliations:** Department of Diagnostic Radiology, School of Medicine, Yale University, 333 Cedar St., New Haven, CT 06520, USA; E-Mails: indukala.doddamane@yale.edu (I.D.);david.cheng@yale.edu (D.C.)

**Keywords:** targeted radiotherapy, radionuclide, radioimmunotherapy

## Abstract

Targeted radiotherapy is an evolving and promising modality of cancer treatment. The killing of cancer cells is achieved with the use of biological vectors and appropriate radionuclides. Among the many advantages of this approach are its selectiveness in delivering the radiation to the target, relatively less severe and infrequent side effects, and the possibility of assessing the uptake by the tumor prior to the therapy. Several different radiopharmaceuticals are currently being used by various administration routes and targeting mechanisms. This article aims to briefly review the current status of targeted radiotherapy as well as to outline the advantages and disadvantages of radionuclides used for this purpose.

## 1. Introduction

The development and evolution of modern chemotherapy during the second half of the twentieth century has improved the clinical outcome of patients with various forms of cancer [1]. Still, in the vast majority of malignancies, the efficacy of systemic chemotherapy is very limited, with 90% of all drug cures occurring in only 10% of cancer types [2].

Unlike systemic chemotherapy, the goal of targeted radiotherapy is the selective delivery of radiation to cancer cells in a way that causes minimal toxicity to surrounding normal tissues. The basis for successful radionuclide therapy is selective concentration and prolonged retention of the radiopharmaceutical within the tumor. Tumor response depends on numerous factors, including cumulative radiation dose delivered, dose penetration, and tumor radiosensitivity. The radiation effect is maximally achieved when the radiation dose is entirely absorbed by the tumor. In practice however, this is almost never the case because of biologic turnover.

## 2. Radiopharmaceuticals and Targeting Mechanisms

The selection of the appropriate radionuclide depends on its nuclear decay properties, specifically, emission characteristics and physical half-life. The treatment of bulky tumors by radionuclides that emit high energy alpha or beta particles is the preferred approach (Tables 1 and 2); however, for the eradication of small clusters of cancer cells or small tumor deposits, radionuclides that emit Auger electrons are considered to be beneficial because of their high level of cytotoxicity and short-range biological effectiveness [3].

## 3. I-131 Treatment for Thyroid Cancer

Treatment of differentiated thyroid cancer (DTC) with radioactive iodine (RAI) is the oldest and most common targeted radiotherapy used in the United States and worldwide. It aims at destroying residual microscopic tumor tissue to reduce the recurrence rate, ablating remaining thyroid tissue to optimize follow-up conditions (e.g., thyroglobulin measurement), and completing staging by a highly sensitive post-therapeutic whole-body scintigraphy [6]. Papillary and follicular thyroid cancers generally express the sodium iodide symporter, which is the key cellular feature for specific uptake of radioactive iodine [7,8].

Following a total or near total thyroidectomy with or without lymph node dissection, RAI ablation of remnant thyroid tissue with I-131 facilitates long term surveillance using serum thyroglobulin measurements. Generally, I-123 whole body scan (WBS) is used prior to therapy to evaluate uptake in the remnant thyroid tissue. This scan can also evaluate the presence of pulmonary and skeletal metastases. The American Thyroid Association (ATA) guidelines published in November 2009, suggest that a pre-therapy I-123 WBS is not necessary for stages I and II or low risk patients since empiric dosing is often not affected by the results [9]. However, based on our clinical experience in our institution, we recommend obtaining an I-123 WBS scan prior to RAI treatment.

According to ATA guidelines, RAI ablation following the initial postoperative management of DTC is recommended for patients with stage III or IV disease, most patients with stage II disease age <45 years and all patients with stage II disease age 45 years or older, selected patients with stage I disease especially those with multifocal disease (≥2 foci, at least one of them >1 cm), nodal metastases, extrathyroidal or vascular invasion, and/or more aggressive histologies (e.g., tall cell, insular, columnar cell carcinoma) [9].

RAI dose is usually determined empirically. Suggested dose for remnant ablation in low risk patients is 30–100 mCi. When residual microscopic disease is suspected or there is more aggressive tumor histology, higher doses (100–200 mCi) may be appropriate. Recent studies suggest that there are no statistical differences in 5 year outcome using low or high doses of I-131 ranging between 30–100 mCi in remnant ablation in low risk DTC patients [10,11]. However, in a study by Kukulska *et al.*, 22% of such patients who initially received 30 mCi required a second course of treatment for complete ablation [10]. One study by Maxon *et al.* suggested that I-131 treatment alone can effectively treat tumor foci that exhibit iodine uptake, as long as a radiation dose of at least 80 Gy is delivered; doses below 35 Gy do not usually bring about a desirable response [12]. Response to I-131 treatment is also dependent on the sensitivity of the tumor to radiation, with younger patients having greater responses as a result of well-differentiated tumors and smaller lesions (<1 cm in diameter) [13].

An alternative approach to fixed dosing is an individualized dosing based on remnant uptake or lesion and bone marrow dosimetry prior to therapy. For this, the European Association of Nuclear Medicine (EANM) Dosimetry Committee recently published a standard operation procedure. General guideline is to deliver tumor doses exceeding 80 Gy and absorbed dose to blood should be below 2 Gy [14,15].

In case of recurrent or persistent DTC, surgical resection is usually considered as the first line of treatment. In this case, RAI therapy is an effective alternative and/or adjuvant treatment option for cure, symptom palliation, and decreasing tumor burden. According to EANM guidelines, nonresectable metastatic lymph nodes, pulmonary micrometastases, nonresectable or incompletely operable macrometastases in the lungs and nonresectable soft-tissue metastases are definite indications for recurrent RAI therapy. Furthermore, optional fields of application include recurrent radioiodine-sensitive lymph node and distant metastases (adjuvant role), nonresectable small and*/*or multiple lymph node metastases, inoperable bone metastases, micrometastases, and anaplastic or poorly differentiated thyroid cancer with relevant well-differentiated portions and thyroglobulin (Tg) expression [16].

Patient preparation prior to treatment is necessary to maximize iodine uptake. In order to achieve elevated serum thyroid stimulating hormone (TSH) levels, patients are either withdrawn from their thyroid hormone replacement or given recombinant human TSH (rhTSH) intramuscularly. In most institutions, patients are advised to follow a low iodine diet for 1–2 weeks before I-131 administration.

Elevated serum TSH level (>30 mU/L) is desirable to increase the sensitivity of detection using imaging modalities or tumor marker Tg, to increase the efficacy of I-131 radioiodine ablation of thyroid remnant in patients who have undergone a total or near total thyroidectomy and for curative or palliative treatment of metastatic thyroid cancer. Patients often experience symptoms of hypothyroidism including fatigue, depression, and decreased cognitive function, which may impact their daily activities significantly. In a study by Pacini *et al.*, patients who were withdrawn had a lower quality of life, and had difficulty performing work because of physical health issues [17]. Traditionally, elevated serum TSH is achieved by hormone withdrawal, which may take 4–6 weeks. The duration of hormone withdrawal symptoms can be shortened with T3 supplement during this period and weaned for only 8–10 days prior to treatment. A similar rise is serum TSH can be achieved within a shorter period from total hormone withdrawal. Alternatively, rhTSH can bypass these symptoms and should be considered for low risk patients. Some studies have shown a lower radiation burden to non-thyroidal tissues when treatment was performed under rhTSH stimulation as compared to hormone withdrawal. Currently, there is insufficient data to recommend rhTSH mediated therapy for all patients with metastatic disease being treated with RAI [9].

Generally accepted selection criteria for RAI treatment in patients with metastases include younger age, well-differentiated tumor histology, high RAI uptake, smaller metastases, location in lungs, stable or slow progressive disease, low uptake of F-18 FDG, and repeated RAI treatment (response rate: 85%, with 96% of complete responses seen with a cumulative activity <600 mCi) [18]. One of the major concerns with high treatment doses compared to relatively lower doses of I-131 for thyroid cancer includes their immediate and long term side effects on salivary glands and oral cavity. In addition, concerns also have been raised for secondary malignancies especially following multiple therapies. However, there has been conflicting data as to whether treatment with I-131 increases this type of risk. Given the lack of comparison or control groups, the reliability of a few published articles in this regard is questionable [19,20]. RAI treatment has been a widely accepted treatment modality in the management of thyroid cancer across the world and will continue to do so, given its effectiveness and safety profile.

## 4. Y-90 Ibritumomab Tiuxetan and I-131 Tositumomab for Lymphoma Treatment

Despite chemotherapeutic advances, numerous patients with low grade lymphoma still die from their disease [21]. Radiolabeled anti-CD20 antibodies Y-90 ibritumomab tiuxetan and I-131 tositumomab have been introduced within the past decade, yielding response rates of 50–80% in patients with diseases refractory to other treatment modalities [22,23]. Ibritumomab is a murine IgG_1_ antibody (Ab) that targets the CD20 antigen. Tositumomab is an IgG_2a_ murine anti-CD20 Ab, but differs from ibritumomab in how it is linked to the radionuclide. Both of these differ from rituximab (Rituxan; IDEC/Genentech), which is a hybrid Ab targeting a cluster of epitopes of the CD20 antigen, except on mature plasma cells. The CD20 antigen is found on the surface of pre–B lymphocytes, mature B lymphocytes and more than 90% of B-cell non-Hodgkin's lymphoma (NHL) [24,25]. The antibody recognizes epitopes in the extracellular domain of the CD20 antigen, and forms on antibody-antigen immune complex, which induces apoptosis, complement-dependent cytotoxicity, and antibody-dependent cytotoxicity [26,27].

Application of nonradioactive antibodies increases the therapeutic window of radioimmunotherapy (RIT) by increasing tumor to background ratio. Monoclonal antibodies (mAb) administered intravenously target cells to promote cell death. However, bioavailability of a small mass quantity of radioactively labeled mAb may be diverted from the tumor to normal B-cells in the circulation or spleen. Administering a pharmacologic quantity of nonradioactive mAb prior to RIT administration will occupy and “mask” the antigen on normal B-cells in the blood and spleen and allow the radioactive antibodies to better access malignant B-cells. This ensures high bioavailability at tumor sites and reduces the radioactivity retained in the body. This concept of pre-targeting follows the principle of radiotracer kinetics in the field of nuclear medicine. The unbound radioactive molecules will clear rapidly from the blood and tissues, thus reduce radiation induced toxicity to normal organs, especially the bone marrow. It will also enhance the tumor-to background ratio and the delivery of a higher therapeutic dose [28].

Y-90 Ibritumomab tiuxetan is a novel radioimmunotherapeutic agent for the treatment of relapsed or refractory low-grade, follicular or CD20^+^-transformed NHL [28]. Y-90 Ibritumomab tiuxetan combines the tumor-specific targeting from mAb with the tumorcidal effect of radiation within a small volume. A large prospective phase III trial by Morschhauser *et al.* showed high efficacy with no unexpected toxicities in the setting of consolidation first-line therapy [29]. This study led to the approval of Y-90-ibritumomab tiuxetan in both Europe and in the United States in September 2009.

In general, both ibritumomab and tositumomab treatments are well tolerated. The most common side effect in patients previously treated with chemotherapy is severe cytopenia due to reversible myelosuppression. Similar efficacy and toxicity profiles are expected from both agents, including the development of human anti-mouse antibodies (HAMA), in patients with an intact immune system in particular. Repeated exposure will increase the risk. Formation of the HAMA-antibody complexes can dramatically reduce the bioavailability and thus targeting efficiency [30]. I-131-Tositumomab differs from Y-90-ibritumomab tiuxetan in that I-131-tositumomab has unlabelled “cold” antibody added in the preparation, whereas Y-90-ibritumomab tiuxetan has “cold” chimeric rituximab as the additive. This may help explain why Y-90-ibritumomab tiuxetan demonstrated HAMA or HACA (human anti-chimeric antibody) rate is 2% [31], while it is significantly higher (10%) in relapsed patients treated with I-131 tositumomab. When I-131 tositumomab was used as first line treatment, the reported incidence was even higher at 65%, attributable to an intact robust immune system [32].

Another major difference between Y-90-ibritumomab tiuxetan and I-131-tositumomab is the method in determining the therapeutic dose. In the United States, a pre-therapy scan with In-111-ibritumomab tiuxetan is used to assess a safe biodistribution of the radioactive Y-90-ibritumomab tiuxetan. Imaging is performed to confirm the expected biodistribution of the radioimmunoconjugate and not used for the assessment of tumor uptake. If pre-therapy biodistribution is acceptable, treatment dose is calculated based on patient's platelet counts and body weight up to a maximum of 32 mCi total dose. Each dose of the radiolabeled antibody administration is preceded by an infusion of rituximab to assure the biodistribution of the radioimmunoconjugate [33].

On the other hand, a dosimetry study is performed to calculate the prescribed dose of I-131 tositumomab. The gamma photon emission from the I-131 allows pre-therapy imaging and patient-specific dose calculation patient-specific maximally tolerated whole body radiation dose taking into account variability in the rate of antibody clearance from each individual [34]. The amount of radioactivity for RIT may be determined by several methods, including those based on whole-body retention and on radiation burden to the limiting organ. The goal of each approach is to deliver maximum amount of radioactivity as tolerated by the critical organs. A retrospective study by Rajendran *et al.*, based on a review of 100 patients' records showed that a dosimetry method based on whole-body retention may underestimate the organ doses. They concluded that a preferable approach may be organ-specific dosimetry [35].

Many studies demonstrated the efficacy of Y-90-ibritumomab tiuxetan and I-131 tositumomab to be similar in patients with chemotherapy- refractory low-grade relapsed NHL, with an overall response rate of 60%–83% and a complete response (CR) rate of 15–52% [31,36-41]. Song *et al.*, using Monte Carlo based dosimetry in patients with lung metastases, found that I-131 may provide a therapeutic advantage over Y-90, especially in tumors with a radius less than 2.0 cm, as well as with a lower tumor burden due to a shorter pathlength of the beta particle from I-131 radionuclide [42]. The biologic effects of radiation therapy may lag behind those of chemotherapy. Jacene *et al.* found that a positive response at 12 weeks after RIT correlated with long-term overall survival and proposed performing the initial assessment at this time point was reasonable. However, a watch-and-wait approach could be taken for those with a partial response (PR), near-CR, or CR, because latent responses may occur beyond 12 weeks after therapy. For patients who have not responded by 12 weeks, alternative therapies may be initiated [43]. PET/CT imaging before and after the treatment has been shown to be useful in the evaluation of treatment response as well as in staging and follow-up of tumors, particularly useful in lymphoma patients [44].

Radiolabeled monoclonal antibodies have been shown to be effective in patients with chemotherapy-refractory, low-grade, or transformed low-grade NHL [32]. Witzig *et al.* demonstrated that patients with indolent histologies, lower tumor grade, fewer than twosites of disease, and less bulky (<7 cm) disease were more likely to respond to Y-90-ibritumomab tiuxetan [39]. On the other hand, Fisher *et al.* showed that advanced age, prior radiotherapy, less than a 75-cGy dose of total-body radiation, bulky disease (≥5 cm), prior anthracycline-containing chemotherapy regimens, and no response to the last chemotherapeutic regimen are predictive of a lower overall response rate [45]. Kaminski *et al.* found that a single course of I-131 tositumomab as initial therapy can induce 75% complete remission in patients with advanced follicular lymphoma, and patients who had both a CR and molecular response, as determined by the lack of BCL2 gene rearrangement in the bone marrow, had a progression-free survival of five years. Their findings suggest that this subset of patients may not benefit from additional or more intensive treatments [23].

Treatment-related myelodysplastic syndromes and acute myeloid leukemia is a well-known delayed complication in patients with low grade NHL [46]. This is thought to be due to repetitive exposure to cytotoxic drugs over a relatively long natural history of the disease. The success of RIT inducing durable remissions in patients with relapsed and refractory NHL can extend the period of observation for bone marrow disease. However, it will be important to separate the effects of RIT from the total incidence of treatment-related myelodysplastic syndromes and acute myeloid leukemia [47].

Lymphoma treatment using RIT appears to be generally well-tolerated and effective in selected groups of patients. Further clinical investigations are needed to increase its applications to other patient groups.

## 5. Peptide Receptor Radionuclide Therapy for Neuroendocrine Tumor Treatment

Somatostatin receptor expression in majority of gastroenteropancreatic neuroendocrine tumors (GEPNET) offers the opportunity for radiopeptide therapy using radiolabeled somatostatin analogues. Radiolabeled somatostatin analogues, such as octreotide, with a high affinity to sub-classes of this receptor have been in use for identification of somatostatin receptor—positive tumors for decades [48]. Clinical peptide receptor radionuclide therapy (PRRT) studies are being performed using different agents. [In-111-diethylenetriaminepentaacetic acid (DTPA)]-octreotide was the first analog introduced [49-52], followed by [Y-90dodecanetetraacetic acid (DOTA), Tyr3]octreotide (Y-90-DOTATOC) [51,53-55], [Y-90-DOTA]-lanreotide [56], and [Lu-177-DOTA, Tyr3]octreotate [57,58].

Radiopeptides incorporating Indium-111, Yttrium-90, or Lutetium-177 have demonstrated both biochemical and radiologic responses in phase I and phase II studies. Despite the potential of this approach, there is still little consensus on the optimal isotope and dose for these agents. Rigorous studies focusing on including inclusion criteria and dosing procedures are needed to better define their role in treatment [59]. Indium-111 emits gamma photons at 172 and 245 keV, which can be used for scintigraphy. Auger electrons, with tissue penetration of 0.02 to 10 μm, and conversion electrons with tissue penetration of 200 to 500 μm, are also emitted. Therefore, it was thought that the high doses of In-111-DTPA may be useful for PRRT. In a phase I study by Valkema *et al.* involving 40 patients treated with In-111-DTPA-octreotide, the efficacy in large neuroendocrine tumors was low, with minimal efficacy in end-stage patients [60].

Bone marrow toxicity with In-111-DTPA-octreotide treatment has been observed at cumulative doses above 100 GBq (2.7 Ci), and hence, this is considered to be the maximal tolerable dose. Valkema *et al.* suggested that dosing beyond this limit should be weighed between an improved quality of life and the risk of severe adverse effects to the bone marrow [60]. Side effects observed by Anthony *et al.* include discomfort at the injection site, thrombocytopenia, anemia, hepatotoxicty, nephrotoxicity, neurotoxicty, and musculoskeletal pain, all of which were transient [61]. However, the lack of response in lower doses and limited toxicity in their patients warranted dose escalation studies. Serious side effects observed by Valkema *et al.* were leukemia and myelodysplastic syndrome in patients treated with total cumulative doses of >100GBq (2.7Ci) [60].Since kidneys express several subtypes of the somatostatin receptor [62], they are at risk for toxicity.[Y-90-DOTA0,Tyr3]Octreotide demonstrated a higher affinity for somatostatin receptor subtype 2 with a greater stability than with an In-111 radionuclide. Different protocols have been used and despite some differences, complete or partial response rates are similar at 10–30%, which is better than In-111-DTPA-octreotide. In a multicenter study by Valkema *et al.*, patients treated with [Y-90-DOTA0,Tyr3] octreotide were given escalating doses up to 14.8 GBq/m2 (400 mCi/m2) in four cycles up to 9.3 GBq /m2 (250 mCi/m2) each dose, without reaching the maximum tolerated single dose [63]. Bushnell *et al.* reported a favorable clinical response as determined by a scoring system, which included weight, patient-assessed health score, Karnofsky score, and tumor-related symptoms, in 14/21 patients treated with a total cumulative dose of 360 mCi [Y-90-DOTA0,Tyr3] octreotide in three cycles [64].

Kwekkeboom *et al.* demonstrated the uptake of radioactivity, expressed as a percentage of the injected dose of [Lu-177-DOTA0,Tyr3]octreotate, was comparable to [In-111-DTPA0]octreotide in kidneys, spleen, and liver, but was found to be three- to four folds higher in four of five tumors with the exception of papillary thyroid carcinoma. This profile can deliver a higher dose to tumors while sparing the dose-limiting organs. Another advantage of Lu-177 over Y-90 is its shorter range of the beta particle in tissue (2 mm versus 12 mm, respectively) [65].

In a study by Kwekkeboom *et al.*, response rate when patients with GEPNET were treated with [Lu-177-DOTA0,Tyr3]octreotate was found to be 46%. This number included the patients with minimal response (MR, defined as >25% but <50% reduction in tumor size) in addition to patients with PR and CR. They also noted a subclass of MR patients with slow growing GEPNET and often with a partly cystic appearance. In fact, the fraction of patients with PR (28%) was greater when compared to CR (16%) or MR (2%) [66].

According to European Neuroendocrine Tumor Society (ENETS) guidelines published in 2009, response to PRRT should be evaluated by a combination of symptomatic and biochemical responses along with imaging response. CT or MRI can be used in follow-up imaging, but neither can provide information on functionality in cystic or necrotic tumors [67].

Up to one-third of metastasized or recurrent differentiated thyroid cancers (DTC) may dedifferentiate over time, characterized by the loss of growth-regulating mechanisms governed by TSH and/or a decline in iodine avidity protecting them from RAI therapy [68,69]. This effect is commonly attributed to a loss or reduced expression of the thyroidal sodium/iodide symporter. Targeted radiotherapy with somatostatin analogues is a potential treatment option for inoperable non iodine-avid DTC. However, intramuscular injection of long-acting octreotide for six months in eight patients with progressive non-iodine-avid but somatostatin receptor-positive thyroid cancer did not yield a significant therapeutic response [70].

Although results with [Y-90-DOTA0,Tyr3]octreotide and [Lu-177-DOTA0,Tyr3]octreotate appear promising, reported varied response rates. Factors limiting direct comparison of many studies include: study design (retrospective), non-randomization of patients, lack of control group, and heterogeneity in treatment population and dosing. Furthermore, variability in response criteria (e.g., imaging, symptomatic relief, as well as hormonal and biochemical responses) made comparison inequitable.

## 6. I-131-Metaiodobenzylguanidine Therapy of Neuroblastoma and Other Neuroendocrine Tumors

For almost three decades, I-131-metaiodobenzylguanidine (MIBG) has been used as a treatment option for NET. It has been shown to be efficacious in chromaffin (neuroblastoma, pheochromocytoma, and paraganglioma) as well as for carcinoid and other less common tumors. Uptake of MIBG, a norepinephrine analogue, in tissue reflects rich adrenergic innervation and/or catecholamine excretion [71-73].

Neuroblastoma is the most common extracranial childhood tumor, and it is very radiosensitive. Most stage I and II patients are treated by surgery alone. Stage III patients are given neoadjuvant chemotherapy prior to surgery. I-131 MIBG therapy is usually considered for stage III and IV patients. In addition to tumor growth control, symptomatic relief can be sought.

Treatment planning involves a staging workup including ultrasonography, CT or MRI studies, FDG-PET/CT, I-123-MIBG scintigraphy, and tissue biopsies. A bone marrow biopsy is also necessary to assess bone marrow failure in neuroblastoma, which is an absolute contraindication for I-131-MIBG treatment. Another absolute contraindication is renal failure, since this will decrease radiotracer clearance and prolong internal radiation exposure. Relative contraindications include incontinence and radiation protection considerations for family members and the public. Life expectancy of the patient to be treated should be greater than one month. Other considerations should include discontinuing medications that might interfere with MIBG uptake, including calcium channel blockers, sympathomimetics, and reserpine. The thyroid gland should be protected with potassium iodide or perchlorate administration prior to treatment.

I-131-MIBG should be infused over a period of at least one hour to avoid the severe acute side effects of the MIBG itself. The infusion system should be shielded to reduce radiation exposure of the staff. In addition, sunlight exposure of the MIBG should be avoided. During the infusion, vital signs have to be closely monitored by the staff or an automatic blood pressure measuring system, since blood pressure changes during the infusion are common. Subsequently, patient should receive a copious amount of intravenous fluid to facilitate excretion and minimize radiation exposure [74]. It should be noted that more than 50% of the given dose will be excreted with urine during the initial 48 hours [75,76]. This is especially important in the pediatric age group to avoid urine contamination in order to minimize the radiation exposure to the staff. Bladder catheterization can be considered for this reason. Acute and subacute side effects include nausea and/or vomiting, thrombocytopenia and less commonly leukopenia. In a phase II study by Matthay *et al.*, a group of patients with refractory neuroblastoma were treated with 18 mCi/kg (0.66 GBq/kg) I-131 MIBG and one third of the patients demonstrated hematopoietic toxicity requiring autologous hematopoietic stem cell support. Nonhematologic high grade toxicity was rare, with 5% of the patients experiencing hepatic, 3.6% pulmonary, and 10.9% infectious complications, as well as 9.7% with febrile neutropenia [77].

Other tumors that may benefit from I-131 MIBG therapy include pheochromocytoma, paraganglioma [78,79], medullary thyroid cancer [80], and carcinoid tumors [81,82] with response rates varying between 30% and 75%. In a retrospective study of 37 patients treated with I-131 MIBG in a total of 116 treatment sessions, the overall survival rate was 85% at five years and 70% at 10 years. However, the authors included patients with metastatic carcinoid tumors, pheochromocytoma, paraganglioma, medullary thyroid carcinoma and the data was independent of the histologic origin of the tumor. None of the patients showed CR, but total percentage of patients showing stable disease during the 32-month follow-up period was 82%, which was similar to patients who received additional therapy combined with I-131 MIBG [83]. Although treatment of NETs with I-131 MIBG appears to be effective in symptomatic relief, further randomized studies are needed to evaluate its usefulness against chemotherapeutic agents.

## 7. Metastatic Bone Pain Palliation

Bone pain caused by skeletal metastases from advanced cancers severely reduces patient quality of life. Approximately 65% of prostate or breast cancer and 35% of advanced lung, thyroid, and kidney cancer patients will experience symptoms [84]. In general, greater than 80% of the cases with bone metastases are associated with prostate and breast cancer [85].

Intravenous administration of Sr-89 chloride, Sm-153-lexidronam, and Re-186-etidronate have been approved in different countries for the treatment of bone pain arising from osteoblastic or mixed osseous metastases. Among these agents, Re-186-etidronate is approved in Europe while the others are approved in the US.

Sm-153-Lexidronam is the most commonly used radiopharmaceutical for bone pain palliation from metastatic disease. The molecule combines Sm-153 with calcium salt of ethylenediaminetetramethylenephosphonic acid (EDTMP). In addition to an energetic beta emission (810 keV), it emits gamma photons of 103.2 keV which make imaging possible.

In a comparison trial by Baczyk *et al.*, with breast or prostate cancer patients, complete pain relief was seen in 40% of women and 40% of men treated using Sm-153, and 25% of women and 33% of men achieved complete relief with Sr-89, which appears to be similarly effective. No pain relief was observed in 20% of these patients [86]. Liepe *et al.* compared the effect of treatment with Re-188-HEDP, Re-186-HEDP, Sm-153-EDTMP, and Sr-89 Chloride. In total, 73% of patients reported pain relief (77% after Re-188, 67% after Re-186, 73% after Sm-153, and 72% after Sr-89; *P* = 0.268–0.846). Overall, fifteen percent of the patients was able to discontinue their pain medications after the treatment [87].

Bone seeking radiopharmaceuticals are effective systemic treatment options in improving the quality of life and decreasing morbidity in patients with painful skeletal metastases. It is less commonly used nowadays due to availability of effective alternative pain management options.

## 8. Treatment of Liver Tumors with Yttrium-90 Microspheres-An Example of Physical Targeting

Liver is a common site for metastases from many primary neoplasms including colorectal carcinoma, pancreatic carcinoma, breast cancer, and neuroendocrine tumors. Tumor burden within the liver will affect the morbidity and mortality. In addition to traditional treatments, liver-directed methods include hepatic arterial infusion chemotherapy, conformal radiation, transarterial chemoembolization, radiofrequency ablation, and radioembolization with Y-90 microspheres [88]. Although the mechanism of delivery involves physical targeting, we have included it since it involves administration of a radiopharmaceutical.

Once the patient is determined to be a candidate for radioembolization with Y-90 microspheres, therapy planning must be carried out. This includes evaluation of target volume for the treatment and assurance of safety from the treatment using morphological imaging studies (CT and MRI), as well as functional imaging modalities (PET/CT and SPECT/CT). Dosimetry and physiologic shunts are assessed from imaging with Tc-99m labeled macroaggregated albumin (MAA) administered into the hepatic artery. Determination of arteriovenous anastomoses or physiologic shunts to the lungs is important in order to avoid radiation pneumonitis [89,90]. If shunting to the lungs is more than 10%, the dose administration must be reduced. If there is a >20% shunt, the treatment with Y-90 microspheres should not be performed [91].

Radioembolization with Y-90 microspheres is a promising treatment option for liver tumors with overall response rates of 27–100% as assessed by clinical and/or FDG-PET criteria [91-94]. Multidisciplinary approach and caution regarding patient selection and treatment preparation is particularly important to avoid serious side effects and toxicity associated with this efficacious treatment.

## 9. Conclusions

In this article we have presented a brief review of commonly used targeted radionuclide therapeutic agents. We believe that changes in targeted radiotherapy will continue to improve alongside of progress in tumor biology research. Many institutions are formulating sophisticated radiopharmaceuticals including antibodies, fusion proteins, peptides and small organic molecules as well as sodium iodide gene transfer into tumor cells for diagnostic and therapeutic purposes.

In the end, the rules in radiobiology should be exploited, in particular, radiosensitivity during G2 and mitotic phases of the cell cycle. Hence, a reasonable approach would be to select a radiolabeled agent with an effective half-life comparable to the doubling time of the tumor cells, which may be estimated from a cell culture derived from a biopsy or less precisely from imaging. Unfortunately, this approach is not practical in using long-lived isotopes due to the dosimetry of radiation burden. An alternative approach is to do serial treatments synchronized to the tumor's native biological clock or with an adjuvant cell cycle arresting agent. Randomized studies are needed to evaluate the effectiveness and practicality of this approach.

## Figures and Tables

**Table 1.** Most commonly used radionuclides for targeted radiotherapy in US [4,5].

**Radionuclide**	**Physical half life**	**Emission type (energy in keV)**	**Range in tissue (mm)**	**Advantages**	**Disadvantages**
I-131	8.04 d	Beta (max 606) and gamma (364)	2.5–3	Availability Low cost Easy labeling	High radiation burden to family members and medical personnels
Y-90	64 h	Beta (max 2280)	12	High-energy beta-emission Prolonged tumor retention	Bone marrow toxicity
Sm-153	46.3 h	Beta (max 807) and gamma (103)	3	Short range in tissue	Bone marrow toxicity
Sr-89	50.5 d	Beta (max 1463)	8	Easy labeling	Long half life Bone marrow toxicity

**Table 2.** Commonly used radiopharmaceuticals for targeted therapy.

**Radiopharmaceutical**	**Targeting mechanism**	**Indications**
I-131 as iodide	Thyroid hormone synthesis	Differentiated thyroid carcinomas
I-131 Tositumomab	CD20 Antigen binding	Non-Hodgkin's lymphoma
Y-90 Ibritumomab tiuxetan	CD20 Antigen binding	Non-Hodgkin's lymphoma
Y-90 microspheres	Intravascular trapping	Liver metastasis Hepatocellular carcinoma
Sr-89 chloride	Calcium analogue	Bone pain palliation
Sm-153 EDTMP	Chemoadsorption	Bone pain palliation
Y-90 Octreotide	Somatostatin receptor binding	Neuroendocrine tumors
I-131 MIBG	Active transport into neuroendocrine cells and intracellular storage	Neuroblastoma Pheochromacytoma Carcinoid Paraganglioma Medullary thyroid carcinoma
